# Comparison of Machine-Learning Classification Models for Glaucoma Management

**DOI:** 10.1155/2018/6874765

**Published:** 2018-06-19

**Authors:** Guangzhou An, Kazuko Omodaka, Satoru Tsuda, Yukihiro Shiga, Naoko Takada, Tsutomu Kikawa, Toru Nakazawa, Hideo Yokota, Masahiro Akiba

**Affiliations:** ^1^R&D Division, Topcon Corporation, Tokyo, Japan; ^2^Cloud-Based Eye Disease Diagnosis Joint Research Team, RIKEN Center for Advanced Photonics, RIKEN, Wako, Japan; ^3^Tohoku University Graduate School of Medicine, Sendai, Japan; ^4^Image Processing Research Team, RIKEN Center for Advanced Photonics, RIKEN, Wako, Japan

## Abstract

This study develops an objective machine-learning classification model for classifying glaucomatous optic discs and reveals the classificatory criteria to assist in clinical glaucoma management. In this study, 163 glaucoma eyes were labelled with four optic disc types by three glaucoma specialists and then randomly separated into training and test data. All the images of these eyes were captured using optical coherence tomography and laser speckle flowgraphy to quantify the ocular structure and blood-flow-related parameters. A total of 91 parameters were extracted from each eye along with the patients' background information. Machine-learning classifiers, including the neural network (NN), naïve Bayes (NB), support vector machine (SVM), and gradient boosted decision trees (GBDT), were trained to build the classification models, and a hybrid feature selection method that combines minimum redundancy maximum relevance and genetic-algorithm-based feature selection was applied to find the most valid and relevant features for NN, NB, and SVM. A comparison of the performance of the three machine-learning classification models showed that the NN had the best classification performance with a validated accuracy of 87.8% using only nine ocular parameters. These selected quantified parameters enabled the trained NN to classify glaucomatous optic discs with relatively high performance without requiring color fundus images.

## 1. Introduction

Glaucoma is a disease that causes progressive damage of the optic nerves, and it is the leading cause of blindness in Japan. The neurodegeneration is irreversible, and patients may not be aware of it until its later stages; thus, early diagnosis and treatment are essential to prevent blindness. The optic disc is the point of exit for all retinal nerve fibers to the brain, and thus, it is important to observe the optic disc in glaucoma management. Besides intraocular pressure, which is an evidenced and treatable influencing factor, glaucoma is considered to be a multifactorial disease; some of these factors are myopia, ocular blood flow, and oxidative stress [1]. However, there are no clear guidelines for the treatments. Nicolela proposed a guideline for identifying a glaucomatous optic disc based on its shape [2]. Nicolela's classification contains four types of glaucoma: local ischemic type (focal ischemic (FI)), age-related hardening type (senile sclerotic (SS)), myopic type (myopic (MY)), and generalized enlargement (GE) [2]. Many studies have shown that this classification is helpful for understanding glaucoma pathogenesis [3–5]. Clinically, doctors always diagnose glaucoma by reading color fundus photos and subjectively identifying the specific optic disc type for glaucoma management. Unfortunately, some doctors have reported unmatched cases that make it difficult to decide further glaucoma treatment. Thus, accurate and objective methods are required for classifying optic discs. Meanwhile, it is necessary to reveal classificatory criteria because a comprehensive classification result should be provided to the doctors for them to accurately decide the course of the clinical treatment.

Machine learning has been used increasingly in medical applications such as computer-aided diagnosis. Because machine learning can determine relationships among input parameters and labels, many studies have used it for classifying glaucoma and healthy eyes [6–8]. However, relevant studies for glaucoma management have not been conducted yet, and more research efforts are required.

In this study, we aim to build a machine-learning classification model for objectively identifying glaucomatous disc-type parameters; then, such disc-type parameters are clinically discussed and compared with doctors' criteria.

## 2. Materials and Methods

### 2.1. Materials

**Figure 1:**
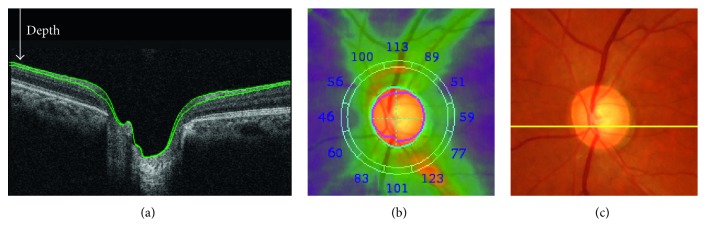
Quantification from optical coherence tomography images. (a) Cross-sectional OCT image at a yellow line in (c), where green lines in (a) show the detected layer information for calculating the retinal nerve fiber layer (RNFL) thickness; (b) RNFL thickness map, where the number indicates the thickness in micrometers in 12 sectors around the optic disc and cyan and magenta circles show automatically detected disc and cup boundaries; (c) a color fundus photo of the optic disc area.

In this study, we recruited 163 eyes from 105 glaucoma patients under a protocol approved by the Institutional Review Board (IRB) (Wako3 26-4). All these eyes were reviewed and classified into four categories by three glaucoma specialists according to Nicolela's definition. With the development of measuring techniques, many methodologies are available for observing the optic disc, such as shape and eye circulation. Compared with color fundus, optical coherence tomography (OCT) based on low-coherence interferometry can image the tissue morphology with micrometer resolution, and therefore, it is being used widely in the ophthalmological field (Figure 1). By using integrated layer analysis software (DRI OCT Atlantis FastMap version 9.30), 48 ocular parameters relevant to the circumpapillary retinal nerve fiber layer thickness (cpRNFLT) and optic disc morphology were quantified [9–11]. The evaluation result against OCT segmentation has been published online as a whitepaper (available at http://www.topcon.co.jp/eyecare/handout) [12].

**Figure 2:**
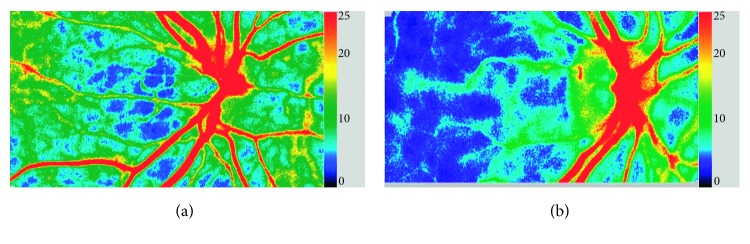
Laser speckle flowgraphy images. LSFG snapshot of (a) a healthy eye and (b) a glaucoma eye. LSFG uses the mean blur rate as an indicator of blood flow. The colormap shows blood-flow-related information in the optic disc with the right-hand-side scale bar, where the blue color indicates lower blood flow and the red color indicates higher blood flow.

Laser speckle flowgraphy (LSFG) allows for the quantitative estimation of blood-flow-related parameters in the optic nerve head by using the laser speckle phenomenon (Figure 2). Thirty-six parameters quantified from its analysis software were also extracted.

Seven demographic parameters, such as gender, age, and spherical equivalent, were also extracted among the 91 quantified ocular parameters for each eye after rudimentary judgement of various ocular parameters, as shown in Table 1.

### 2.2. Feature Selection

In machine learning, feature selection (FS) helps to (1) improve the classification performance by avoiding overfitting, (2) build a time-saving model, and (3) make the built model more understandable to humans. FS methods can be categorized into three types depending on their selection mechanism: filters, wrappers, and embedded types. Filters use general characteristics such as correlation to remove irrelevant features without using any machine-learning algorithms. Minimum redundancy maximum relevance (mRMR), one type of a filter method, is based on mutual information; it has been widely used recently because it assesses the trade-off of maximizing the relevance between each feature and label and minimizing the feature redundancy [13]. Wrappers use classifiers to evaluate the performance and to search for the best combination of features. Embedded methods are quite similar to wrappers in that they also use a machine-learning model; however, they differ from wrappers in that they perform FS as a part of the machine learning. In wrappers, a heuristic search shows higher performance but is too time-consuming, especially for a large number of features. Thus, instead of brute-force selection, more efficient strategies have been developed, such as genetic-algorithm-based feature selection (GAFS) using randomness that mimics natural evolution [14]. Filters are often used in combination with heuristic wrappers for principal selection [15]. In this study, we used a hybrid FS scheme that combines mRMR and a genetic-algorithm-based method. We also applied gradient boosted decision trees (GBDT), which is an embedded method, to compare the effects of the FS schemes.

### 2.3. Machine-Learning Classifiers

Various classifiers have been used to compare different FS schemes in detail. Naïve Bayes (NB) is a simple probabilistic classifier based on Bayes' rule. NB considers all features to be independent of the probability of a label. Support vector machine (SVM) is a supervised machine-learning algorithm that transforms the feature space to a much higher dimension by using kernel functions and finds a linear boundary to achieve the maximum margin between two classes. In this study, we explored SVM with radial basis function (RBF) kernels. A neural network (NN) models the neurons and synapse of the brain, and it enables problems to be processed nonlinearly by identifying the correlation between features and labels.

In this study, we explored the effect of FS on the above three classification classifiers for GAFS. Separately, GBDT, decision trees as the weak learner capable of calculating the feature importance, are applied for comparison with the GAFS schemes.

### 2.4. Proposed Approach

**Figure 3:**
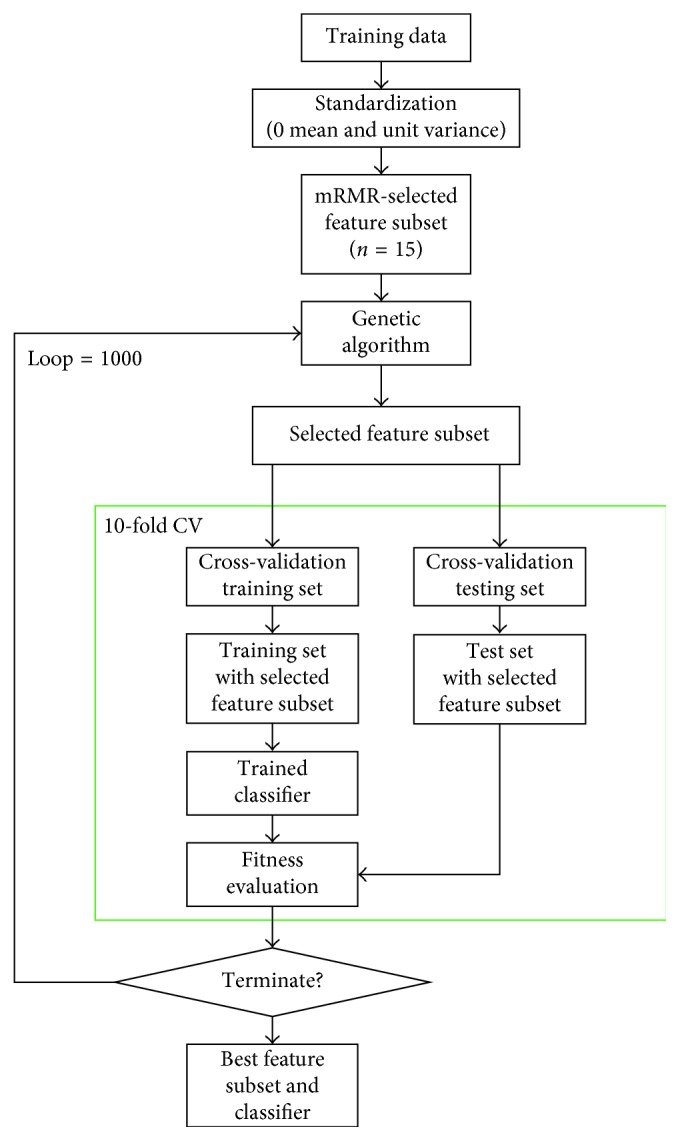
Flow chart of the proposed approach (GAFS).

First, we divided all the eyes into two groups: training data (*n*=114) and test data (*n*=49). With training data, mRMR was first used to find the candidate features (15 features), and then, GAFS with NB, SVM, and NN was applied to find the most valid features and classifiers. To compare the performance of FS and machine-learning classifiers, we used Cohen's kappa of 10-fold cross-validation (CV) for training data as the evaluation criteria (Figure 3). GBDT was trained with the training data without using mRMR and also evaluated with Cohen's kappa of 10-fold CV. Finally, all the developed models were validated by using the test data.

Table 2 lists the parameters used in GAFS.

## 3. Results and Discussion

**Figure 4:**
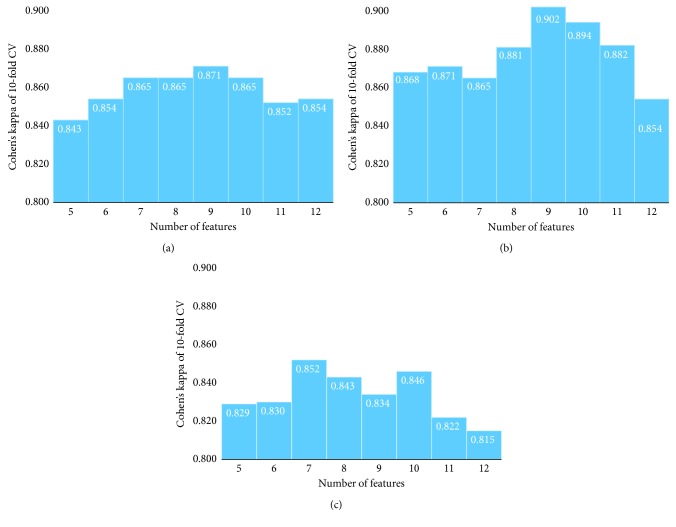
Performance change of classification using different numbers of features. (a) Support vector machine; (b) neural network; (c) naïve Bayes.

Table 3 shows the top 10 contributing quantified parameters ranked by GBDT. Cohen's kappa of 10-fold CV is 0.83 (Figure 4).

**Figure 5:**
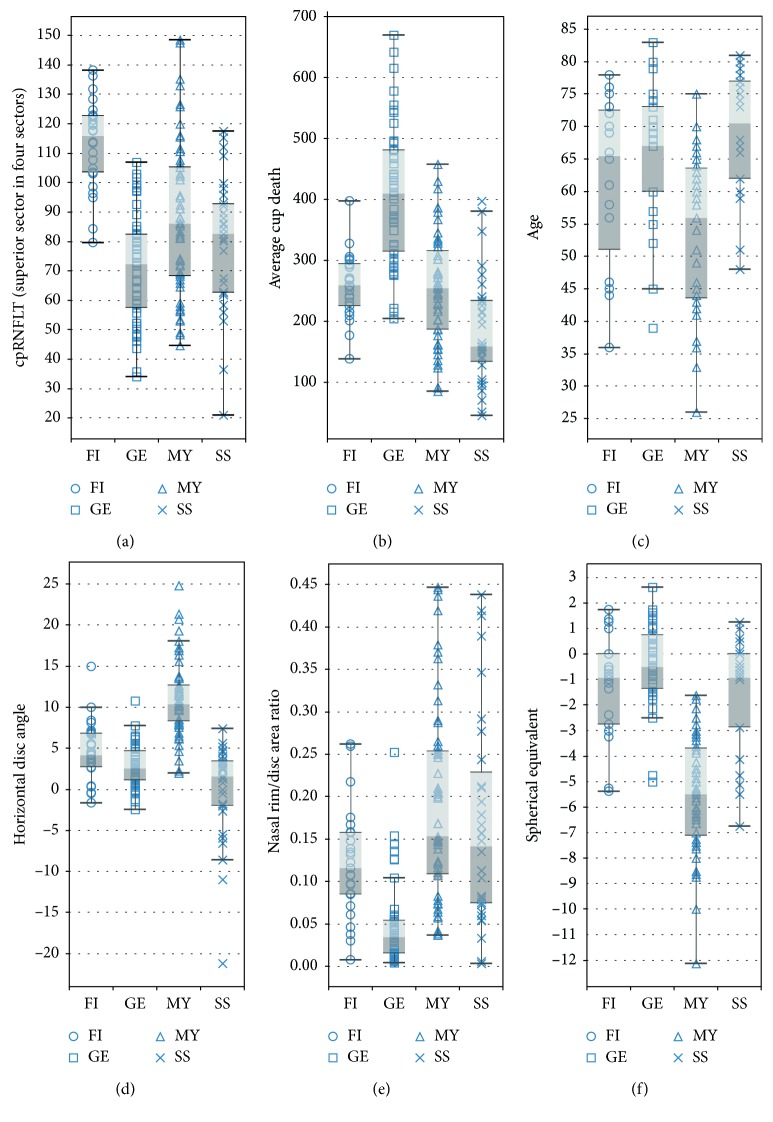
Box-and-whisker plots of common features: (a) cpRNFLT (superior sector in the four sectors); (b) cup area; (c) age; (d) horizontal disc angle; (e) nasal rim/disc area ratio; (f) spherical equivalent.

The best Cohen's kappa of 10-fold CV for SVM, NB, and CV was 0.871, 0.852, and 0.902, respectively, which are better than that of GBDT. Furthermore, Table 4 lists the results of the selected features for each model. This table shows the common parameters for three classifiers, such as age, spherical equivalent, nasal rim/disc area ratio, horizontal disc angle, average cup depth, and cpRNFLT (superior sector in four sectors), which are the six most contributing features calculated by GBDT. Our new classifier shows higher accuracy for such a classification compared to the regression model with Cohen's kappa of 0.73 that was demonstrated in a previous study [10].  Figure 5 shows the box-and-whisker plot of the common features, and these features appear to help discriminate different optic discs, consistent with previous clinical findings. Generally, MY disc type has a low spherical equivalent and tilts resulting in a high horizontal disc angle; it is associated with the onset of glaucoma at a younger age. On the contrary, GE discs generally have a thin nasal rim and a large average cup depth and cup area, while SS discs have a small average cup depth and are associated with the onset of glaucoma at an older age. FI discs showed thickening of the cpRNFL in the superior sector in the four sectors [11].

**Figure 6:**
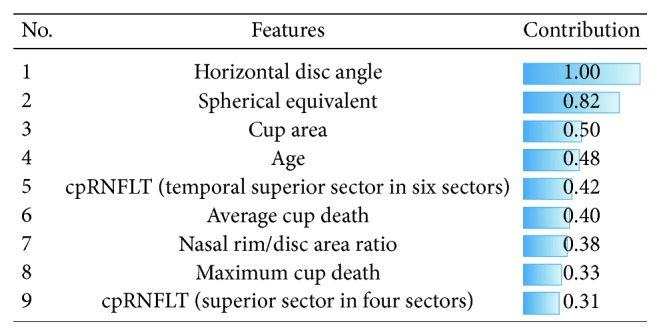
Contribution of each selected feature to Nicolela's classification. Selected features (9 features) when using the NN were sorted by the contribution calculated with the weights of each unit in the NN. The horizontal disc angle had the highest contribution for classifying optic discs.

The NN with just a single hidden layer (number of units: 8) was the best classifier for this problem, in which the nine most valuable ocular parameters were chosen by hybrid FS (Figure 6). Seven parameters (horizontal disc angle, cup area, cpRNFLT (temporal superior in six sectors), average cup depth, nasal rim/disc ratio, maximum cup depth, and cpRNFLT (superior in four sectors)) were extracted from OCT, and two parameters (spherical equivalent and age) pertained to patients' demographic data. This shows the possibility of performing this classification by using only the OCT data set. The contribution of each selected parameter was also calculated by using the weights for each unit in the trained NN (Figure 6) [16]. Doctors can classify and check optic disc types using these features along with their contribution values and not just by reading the color fundus images.

In this study, two additional experiments were performed to validate the current classifier. First, the selected features were investigated by comparing the results obtained from the combination of mRMR and brute-force selection. In brute-force selection, all possibilities are tried one after another until the best accuracy is obtained. As a result, we obtained the same classification parameters and performance as those of the hybrid FS used in this study. Even though the same results were obtained, it should be noted that the calculation time of brute-force selection was ∼120 times that of the proposed method. Second, all data were shuffled to regenerate the training data and test data randomly. We found that the hybrid FS had the highest classification performance in the NN (Cohen's kappa: 0.902) with a different combination of features (*n*=9) in which six ocular parameters, namely, spherical equivalent, cup area, maximum cup depth, average cup depth, cpRNFLT (superior sector in four sectors), and horizontal disc angle, were the common features before and after the shuffling of data. Instead of cpRNFLT (temporal superior sector in six sectors), nasal rim/disc area ratio, and age, the hybrid FS found three new features, namely, RPE height difference, superior nasal rim/disc area ratio, and cup/disc area ratio. Because unselected features after shuffling have high correlations with any feature in the new feature combination, the classification performance did not decrease significantly; for example, the correlation value of the nasal rim/disc area ratio and superior nasal rim/disc area ratio was 0.864, whereas that of the horizontal disc angle and RPE height difference was 0.804.

**Figure 7:**
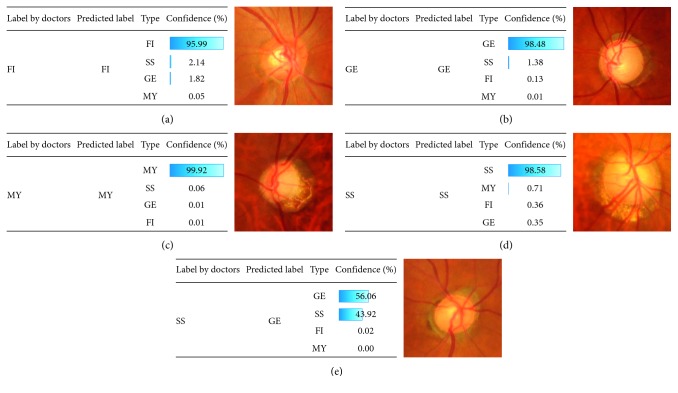
Prediction examples obtained using the NN: (a) successful example of prediction for FI and color fundus photo, (b) successful example of prediction for GE and color fundus photo, (c) successful example of prediction for MY and color fundus photo, (d) successful example of prediction for SS and color fundus photo, and (e) failure example of prediction and color fundus photo.

The proposed model can calculate the confidence of the prediction (Figure 7). When validating the prediction with the test data by using the highest one as the prediction, the overall accuracy was 87.8%. With regard to failure prediction examples, we found that the developed classification model classified the correct answer as the second choice in most cases (Figure 7(e)). If the second choice is also considered to be correct, the accuracy was 95.9%. In some cases, specialists also narrow down the answer to two or more, such as FI and MY optic discs (Figure 7(e)) because FI optic discs clinically always have myopic characteristics as do the MY type. Thus, our machine-learning classification model might well reflect the actual clinical problem, and the prediction calculated by this approach can assist doctors in understanding the glaucomatous optic disc shape among glaucomatous subjects.

There are some limitations in this study. First, we did not use deep learning, which is widely used in classification of affected and healthy eyes with clinical images [17–19]. Deep learning is being recognized as a powerful method for automatically designing effective features directly from the images, when the data are sufficient, which could not be accomplished in this study unfortunately. Second, a single NN with just one hidden layer may not yield good performance. In the future, we will try to combine features learned by the deep learning approach from OCT, LSFG, and color fundus images with the quantified parameters and increase the hidden layers to improve the performance after collecting more data.

## 4. Conclusions

The results show that the proposed approach can objectively classify the glaucomatous optic disc shape with FS and NN by using quantified ocular parameters obtained from ophthalmic examination equipment. The confidence of each predicted optic disc type and the obtained contributing ocular parameters can assist in daily clinical glaucoma treatment.

## Figures and Tables

**Table 1:** Extracted ocular parameters.

No.	Quantification data	Features
1	Patient's background data	Gender
2		Age
3		Spherical equivalent
4		Mean deviation
5		Pattern standard deviation
6		Internal ocular pressure
7		Central corneal thickness

8	Optic disc shape parameters obtained from OCT	Disc area
9		Cup area
10		Rim area
11		Vertical disc diameter
12		Horizontal disc diameter
13		Vertical cup/disc diameter ratio
14		Horizontal cup/disc diameter ratio
15		Cup/disc area ratio
16		Rim/disc area ratio
17		Maximum cup depth
18		Average cup depth
19–24		Average rim/disc area ratio (six sectors)
25		Rim decentering area ratio
26		Horizontal disc angle
27		Disc height difference
28		Retinal pigment epithelium (RPE) height difference
29		Disc tilt angle

30	cpRNFLT average thickness obtained from OCT	Average cpRNFLT
31–34		cpRNFLT (quadrants)
35		Difference in cpRNFLT (superior and inferior in four sectors)
36–41		cpRNFLT (six sectors)
42		Rim decentering cpRNFLT ratio
43		Difference in cpRNFLT (temporal superior and temporal inferior in six sectors)
44–55		cpRNFLT (clockwise sectors)

56	Ocular blood flow parameters obtained from LSFG	Average in all (tissue)
57–60		Average in quadrants (tissue)
61		Skewness in all (tissue)
62–65		Skewness in quadrants (tissue)
66		Blowout score in all (tissue)
67–70		Blowout score in quadrants (tissue)
71		Blowout time in all (tissue)
72–75		Blowout time in quadrants (tissue)
76		Rising rate in all (tissue)
77–80		Rising rate in quadrants (tissue)
81		Flow acceleration index in all (tissue)
82–85		Flow acceleration index in quadrants (tissue)
86		Acceleration time index in all (tissue)
87–90		Acceleration time index in quadrants (tissue)
91		Average ratio of blood stream

**Table 2:** Parameters used in GAFS.

GAFS parameter	Value
Population size	20
Crossover probability	0.7
Mutation probability	0.2
Selection type	Tournament of size 2
Number of generations	1000
Early stopping	Used

**Table 3:** Feature importance calculated by GBDT (top 10).

No.	Features	Feature importance
1	Horizontal disc angle	1.000
2	Spherical equivalent	0.723
3	Average cup depth	0.427
4	Nasal rim/disc area ratio	0.284
5	Age	0.145
6	cpRNFLT (superior sector in four sectors)	0.136
7	cpRNFLT (temporal superior sector in six sectors)	0.127
8	Cup area	0.040
9	Maximum cup depth	0.038
10	Superior nasal rim/disc area ratio	0.038

**Table 4:** Selected features using different classifiers in GAFS.

	SVM	NB	NN
Cohen's kappa	0.871	0.852	0.902

Common features	Age		
	Spherical equivalent		
	Nasal rim/disc area ratio		
	Horizontal disc angle		
	Average cup depth		
	cpRNFLT (superior in four sectors)		

Individual features	cpRNFLT (temporal superior in six sectors)	Disc horizontal diameter	cpRNFLT (temporal superior in six sectors)
	Maximum height difference		Cup area
	Horizontal disc diameter		Maximum cup depth
